# More Than Just Tumor Destruction: Immunomodulation by Thermal Ablation of Cancer

**DOI:** 10.1155/2011/160250

**Published:** 2011-12-29

**Authors:** Sebastian P. Haen, Philippe L. Pereira, Helmut R. Salih, Hans-Georg Rammensee, Cécile Gouttefangeas

**Affiliations:** ^1^Abteilung II fuer Onkologie, Haematologie, Immunologie, Rheumatologie, und Pulmologie, Medizinische Universitaetsklinik, Otfried Mueller Straße 10, 72076 Tuebingen, Germany; ^2^Abteilung Immunologie, Interfakultaeres Institut fuer Zellbiologie, Auf der Morgenstelle 15, 72076 Tuebingen, Germany; ^3^Klinik fuer Radiologie, Minimalinvasive Therapien, und Nuklearmedizin, SLK-Kliniken Heilbronn, Am Gesundbrunnen 20-26, 74078 Heilbronn, Germany

## Abstract

Over the past decades, thermoablative techniques for the therapy of localized tumors have gained importance in the treatment of patients not eligible for surgical resection. Anecdotal reports have described spontaneous distant tumor regression after thermal ablation, indicating a possible involvement of the immune system, hence an induction of antitumor immunity after thermoinduced therapy. In recent years, a growing body of evidence for modulation of both adaptive and innate immunity, as well as for the induction of danger signals through thermoablation, has emerged. Induced immune responses, however, are mostly weak and not sufficient for the complete eradication of established tumors or durable prevention of disease progression, and combination therapies with immunomodulating drugs are being evaluated with promising results. This article aims to summarize published findings on immune modulation through radiofrequency ablation, cryoablation, microwave ablation therapy, high-intensity focused ultrasound, and laser-induced thermotherapy.

## 1. Introduction

The local application of high or low temperatures is frequently used to induce protein denaturation, tissue necrosis, and tumor destruction in order to curatively or palliatively treat localized primary or secondary tumors [1]. Thermal ablative procedures in clinical practice comprise radiofrequency (RF) ablation, microwave ablation therapy (MWA), high-intensity focused ultrasound (HIFU), and laser-induced thermotherapy (LITT) with the use of high temperatures, as well as cryoablation with induction of low temperatures. Primarily all these techniques were applied for the palliative treatment of patients not eligible for surgical resection or frail patients with a reduced functional reserve capacity and many comorbidities [2, 3]. Local thermal ablative methods present several advantages as compared with surgery which include less damage to surrounding healthy tissue, greater patient comfort, for example, less pain and limitation in exercise due to wound healing, improved cosmetic results, and—in times of critical financial situations in the medical facilities—reduced cost and shorter periods of hospitalization [2, 4]. For selected patients, local thermoablative techniques have similar clinical outcomes as compared with historical controls of surgical resection [5–8]. However, except for early hepatocellular carcinoma, no large randomized clinical trial has been performed to directly compare thermoablation and surgical resection so far [9]. In clinical routine, thermal ablation techniques have gained further importance in the treatment of small tumors as an alternative to surgical resection. Their application is limited by the size of the tumor lesions since large tumors (>4 cm) require more expanded treatment with an increased rate of complications and local recurrence [10, 11].

The choice of the most suitable thermal ablation modality depends on different premises. Tumors located in tissues with a high impedance like lung or bone can be better treated with cryoablation or MWA [12–14]. Other factors for the assignment to an ablation modality depend on patient characteristics and comorbidities, on the physician's choice and availability of a certain method in a respective hospital, as well as on tumor location and relative position to other anatomic structures [1]. The clinical indications and characteristic features of each technique are summarized in Table 1.

The concept of thermal treatment for cancer is not new. The first patients with cerebral tumors were already treated with RF ablation in the early 20th century, but it took until the 1990s for RF ablation to become an accepted, commonly used treatment option for primarily unresectable tumor lesions in liver, kidney, bones, and lung [15]. During RF treatment, one or more RF applicators are placed in the target tissue and high-frequency alternating current is generated, leading to frictional heating above 60°C up to 100°C inducing coagulative necrosis [2, 16]. Higher temperatures would result in desiccation and subsequent increase in tissue impedance which limits further conduction of electricity into the tissue [12]. Recent studies have shown that the clinical outcome after RF ablation is comparable or even better in comparison to that of surgical resection. Consequently, RF ablation is currently being discussed as a possible new standard for elimination of metastatic liver lesions and oligofocal hepatocellular carcinoma (HCC) [5, 8, 17] and further as a curative treatment option in HCC and metastatic stages of colorectal carcinoma (CRC) when combined with surgery [18, 19]. Early-stage non-small cell lung cancer (NSCLC) can also be successfully treated with RF ablation. However, retrospective comparative analyses of survival have shown a strong tendency to increased survival benefits for NSCLC patients treated with surgery compared to RF ablation (46 versus 33 months, *P* = 0.054) [20], limiting the application of RF ablation to patients with contraindications against surgery. 

MWA represents a relatively new technique using electromagnetic waves to induce high temperatures of up to more than 100°C. Here also, an active microwave antenna is placed into the tumor. Since MWA does not require the conduct of electric current, temperatures above 100°C do not result in a decline of therapeutic efficacy [12]. This method could therefore be effectively applied in tissues with higher impedance like lung and bone [12]. In humans, MWA is currently mainly applied for the treatment of HCC [21, 22].

During HIFU, ultrasound beams of high energy are applied to focus acoustic energy on a well-defined region inducing tissue vibration. Although single ultrasound beams can penetrate tissue without causing significant heat, focussing beams from multiple directions into a selected region results in a temperature rise to over 60°C and subsequently in coagulative necrosis [23, 24]. HIFU also induces acoustic cavitation which represents an additional mechanical mechanism of tissue destruction. Acoustic cavitation (the expansion and contraction of gaseous nuclei in cells through acoustic pressure) leads to collapse of mitochondria, endoplasmic reticulum, as well as nuclear and cell membranes [25]. This procedure is the only noninvasive thermal technique and allows real-time imaging of the treatment progress by ultrasound (US) [25]. However, the clinical application of HIFU is limited since the size of the multidirectional ultrasound focus is confined by technical boundaries and a treatment time as short as possible is required for an accurate ablation [24]. HIFU has been applied for the treatment of breast, liver, pancreas, kidney, bone, prostate, and soft-tissue tumors [25–27]. 

Laser fibers placed into a tumor lesion are used for laser ablation where photon energy conduction induced heating can reach temperatures of over 50°C. The tissue penetration depth of laser light is only 0.4 mm which implies that multiple laser fibers have to be positioned into a tumor to ensure optimal tissue destruction [28]. However, this limited penetration can facilitate the monitoring and accuracy of the ablation. This technique is experimentally used for the treatment of breast, brain, liver, bone, and prostate tumors [29, 30]. More extensively used is the clinical exertion of laser photocoagulation in retinal diseases, here also leading to retinal scarring [31].

In contrast to all the techniques mentioned above, cryoablation utilizes not high, but extremely low temperatures that sink to −160°C [32]. Cryoablation involves the evaporation of liquid gases and is a purely thermal process which does not require application of electrical current, leading to a broader applicability in high impedance tissues like lung or bone. The extent of tissue destruction can be easily monitored by direct monitoring of *ice-ball *formation with all conventional imaging modalities [1]. Cryoablation is used in broad clinical application, even for the treatment of retinoblastoma in children [33]. In 1–6% of cases cryoablation causes a systemic inflammatory response syndrome (SIR), termed as the *cryoshock phenomenon* which represents a potentially life-threatening complication [34–38] and limits its clinical application, especially for liver tumors [39].

The observation that spontaneous regression of untreated tumors can occur after thermoablation of distant tumor masses may indicate an involvement of immune activation upon thermoablation [39–42]. The initiation, maintenance, and termination of an effective antitumor immune response requires a complex interplay between cellular (immune cells including effector and regulatory subsets) and humoral components (cytokines, chemokines, antibodies). Various constitutive or inducible danger signals released by injured cells are known to play a determinant role in alarming the immune system against self-damage. In this *danger model*, cells dying by physiological processes such as apoptosis will be rapidly eliminated and ignored by the immune system whereas necrotic cells releasing their content in the extracellular space will trigger an immune response [43, 44]. In particular, heat shock proteins (HSP) constitute a group of molecular chaperones which stimulate the maturation of dendritic cells (DC) and carry antigenic peptides from their cells of origin inducing subsequent priming of antigen-specific T cells [45–48]. Local ablative treatment induces necrosis which may naturally modulate all of these parameters by inducing inflammatory processes finally leading to the development of an antitumor specific immune response. A growing series of reports describing inflammatory responses, antigen release and uptake by professional APC and antitumor adaptive immunity shows that this can indeed be the case. This review aims to summarize findings on the modulation of the immune system through high- or low- temperature-induced thermal tissue ablation of cancer in animal tumor models and cancer patients. The respective techniques are presented in the order of common clinical use.

## 2. Radiofrequency Ablation

RF ablation has the broadest application in cancer treatment. It is therefore not surprising that most recent data relating to the activation of the immune system through thermoablation have been obtained using this method (Table 2).

### 2.1. Cytokines and Stress Response

Several groups have evaluated the systemic release of cytokines, chemokines, and various stress factors after RF ablation. Serum levels of proinflammatory cytokines like interleukins IL-1 *β*, -6, and -8, as well as TNF were found to be either increased [51–54] or unchanged [133, 134]. In general, changes were modest and transient (several hours to days after ablation) [38, 52, 53, 134]. Moreover, IL-10 could be elevated in the serum postinterventionally [54, 133]. Over all, no case of severe SIR with multiorgan failure and coagulopathy, but significant increases in body temperature, mean arterial blood pressure, and concomitantly increased serum levels of adrenaline, nor adrenaline or C-reactive protein (CRP) have been reported shortly after RF ablation [53, 54, 133, 134]. 

In murine models, RF ablation induced strong upregulation of mRNA and/or protein levels of HSP-70, HSP-90, and glycoprotein 96 (gp96) as well as translocation of nuclear high-mobility group protein B1 (HMGB1) into the cytoplasm of tumor cells and into the intercellular space [55–57]. More specifically, increased HSP-70 expression was shown to occur at the margin of the ablation zone, the so-called transition zone, both in animals [55, 58, 59] and in human liver cells *in vivo *[62]. The time frame of maximal HSP-70 expression is described to be no more than 24 hours after ablation [56, 60, 62], the protein remaining upregulated in the necrosis surrounding tissue three days after RF ablation [61]. Several factors may influence local expression of heat shock proteins after RF: in rats receiving thermal ablation in different zones of the liver, the degree of HSP-70 expression was observed to be dependent on the relative spatial position of the ablated area to larger liver vessels since the blood stream of these vessels can nourish adjacent cells preserving cellular metabolism and, hence, expression of HSP in these cells [60]. Further experiments in nude rats transplanted with human HCC also suggested a correlation between applied energy and level of expression of HSP-70 and -90 [55]. As we recently described, a significant systemic release of HSP-70 into the serum can also be detected one day after treatment in RF-treated cancer patients, but serum levels did not correlate with ablation volumes, histological tumor type, and other clinical or laboratory parameters [63].

### 2.2. Cellular Immunity

#### 2.2.1. Changes in Peripheral and Intratumoral Immune Cell Subsets

Postinterventional changes in peripheral leukocyte subsets have been observed by several groups and taken as evidence for the immune modulatory effect of RF ablation. Of note, antibody tools for cell subset identification, timepoints of observation, and patient cohorts differed between published studies. A decrease of circulating CD4^+^CD25^+^Foxp3^+^ regulatory T cells (T_reg_) was observed in patients 1 month after RF ablation of lung nodules [54]. In another study including 20 HCC patients, no significant changes in T-cell subsets were detected 1 month after RF (naïve or memory CD4^+^, CD8^+^) while increased percentages of activated T cells and circulating NK cells were noted in randomly selected patients from the study cohort [70]. The same group later described a marked expansion of CD3^−^CD56^dim⁡^ effector NK cells 1 week and 4 weeks after treatment [67]. Matuszewski and colleagues evaluated lymphocyte subpopulations after RF ablation of renal cell carcinoma (RCC) in 6 patients and found a globally increased proportion of activated T cells in the majority of patients (CD3^+^HLA-DR^+^) whereas effects on CD4^+^, CD8^+^, and NK (CD56^+^CD16^+^) cells varied among individuals and at different timepoints [71]. In patients with colorectal liver metastases, but not with HCC, a transient decrease in CD3^+^CD4^+^ T cells was noted shortly (day 2) after treatment [75]. Although these and further observations are heterogenous, they collectively suggest an impact of RF ablation on various peripheral cell subsets, including T and NK cells [61], but also neutrophils, monocytes, B lymphocytes, and even DC [51, 54, 66, 75]. 

The assessment of tumor-infiltrating cells before and following RF ablation is intrinsically difficult in patients and available data have been obtained in various animal models. Most reports describe infiltration of immune cells in the transition zone hours to days after treatment. Granulocytes, macrophages, plasma cells, DC, CD3^+^, and CD4^+^ cells were found [49, 64, 68]. Interestingly, neutrophils and lymphocytes could also infiltrate distant, untreated metastases [65].

#### 2.2.2. Antitumor Specific Responses

Few data addressing the adaptive immune response to tumors after RF ablation are available. 

In a transplant-tumor model of VX2-hepatoma, rabbits were randomly assigned to treatment with RF ablation or to observation. Two weeks after RF ablation, the activation of tumor-lysate specific T cells was detected and persisted over a postinterventional observation period of 6 weeks [68]. Animals in the RF-treated group had a significant survival increase [68]. 

Antigen-specificity of RF-induced antitumor T-cell responses was investigated in several reports. Dromi and coworkers used a murine urothelial carcinoma expressing the male minor histocompatibility antigen HY which was inoculated to female mice. T-cell responses against MHC-class I and class II HY-derived epitopes were significantly increased in the group of mice having received tumor RF ablation as compared to control animals. This was accompanied by an enhanced control of tumor growth, including upon rechallenge [49].

In a mouse model of OVA-expressing melanoma, adoptive transfer of splenocytes from RF-treated to naïve mice led to a growth retardation of OVA^+^-, but not OVA^−^-tumors after rechallenge, and to complete tumor elimination in 20% of the mice. The treatment could also induce long-lasting immunity since RF-treated mice surviving the first tumor inoculation were completely protected after a second challenge 70 days later [72]. Moreover, intratumor injection of tagged-OVA led to antigen uptake and maturation of CD11c^+^ cells in the tumor-draining lymphnode, albeit to a lesser extent than after cryoablation which was directly compared to RF ablation in this model [69].

In patients, HCC-reactive T cells were detected with IFN*γ* ELISPOT in 4/20 patients before RF ablation upon stimulation of PBMC with lysate of autologous tumor cells obtained either before or after treatment. One month after RF treatment, cellular reactivity was observed in 9/20 patients, strongly suggesting an *in vivo* immunization effect after RF-intervention [70]. Similar results were reported in two further cohorts of HCC and CRC patients [76].

Three recent publications have addressed the antigen-specificity of the RF-induced T-cell responses in patients. Napoletano and colleagues detected an increased IFN*γ* production upon stimulation with MUC-1-derived glycopeptides in 2 patients treated for liver metastases and also an increase in circulating CD3^−^CD19^+^ B cells. However, the specificity of antibodies was not studied [75]. Hiroishi and coworkers investigated CD8^+^ T-cell responses against MAGE-1, NY-ESO-1, and GPC3 antigens in patients with HCC and found that antigen-specific T cells were already detectable in samples obtained before RF ablation, and increased in approximately half of the patients [77]. Recently, we evaluated the occurrence of tumor-antigen specific T cells or antibodies after RF ablation in 55 cancer patients and found an increase in antigen-specific antibodies, and CD4^+^ or CD8^+^ T cells in several individuals receiving RF ablation alone or shortly after chemotherapy [78].

#### 2.2.3. Combination Therapies

All the results presented above show that RF ablation is able to induce tumor-directed immunity; however, the observed therapeutic effects are limited. Combination therapies have therefore been already tested in preclinical models, with the aim to enhance antitumor responses and protection. For OVA-expressing melanoma, CTLA-4 blockade or T_reg_ depletion (with anti-CD25 mAb) showed improvement in tumor control and enhanced induction of OVA-specific CD8^+^ T cells whereas CTLA-4 mAb application without RF ablation did not mediate the same effects [72].

The coadministration of the monocyte attracting chemokine ligand 3 and inflammatory protein-1*α* (CCL3/MIP-1*α*) [73], antibody-conjugated IL-2 [74] or even chemotherapy (liposomal doxorubicin) [59] also enhanced the effects of RF ablation. Finally, whereas intratumor injection of unloaded DC did not synergize with RF-treatment, application of tumor-lysate loaded DC was reported to abrogate tumor relapse in most animals. Interestingly, while vaccination with DC alone was ineffective with regard to survival benefits, the combination with RF ablation significantly improved the survival of tumor bearing mice [49, 57].

All these reports provide a strong rationale for testing the combination of RF therapy with immune-modulating agents in cancer patients. It has to be noted that—besides RF ablation—many patients currently receive additional therapies like chemotherapy which may also influence the development of tumor-specific immune responses as recently recognized [137].

### 2.3. Immune Response and Clinical Course

The relationship between occurrence of antitumor immunity after RF ablation and clinical outcome still remains elusive. In HCC patients with induced tumor-specific T-cell reactivity after RF ablation, the local- and distant-site recurrence was similar [70]. In contrast, Hiroishi and colleagues observed a correlation between the frequency of tumor-antigen specific T cells and a favorable tumor-free survival in HCC patients [77]. Here, it has to be noted that patients could additionally receive transarterial chemoembolization (TACE). We have recently observed a tendency to a better survival for patients who presented with at least a twofold increase of HSP-70 in the serum one day after treatment [63]. Since patient cohorts were small in all three studies, results need further confirmation.

## 3. Cryoablation

### 3.1. Special Premises of Cryoablation

While thermal techniques utilizing lethal high temperatures have been so far mostly described to stimulate immune responses, cryoablation has been described to exert both stimulatory and suppressive effects on the immune system. These particular features could be due to the specific physiological mechanisms of cold injury including (i) direct cellular damage through formation of ice crystals, and (ii) vascular and endothelial injury with potential ischemia [82]. Whereas most other thermoablative techniques are believed to induce essentially coagulative necrosis, apoptotic cells might be also present at the outer rim of the ablation zone after cryoablation. According to the *danger model*, apoptotic cells do not release their cellular content (antigens, HSP, and HMGB1) and induce immunological tolerance [43, 44]. It has been proposed that larger numbers of apoptotic cells might cause tissue protection and lead to immunosuppression while larger numbers of necrotic cells could serve as immunostimulators [4]. More recently, they showed that the cryoablation modality itself, that is, rate of freeze, influences both tumor growth and T cell recruitment [79]. Moreover, technique and rate of freezing cycles could play a role in the precise mechanisms of the watershed between immunosuppression and immunostimulation after cryoablation [79]. However, this model is questioned by more recent reports showing that apoptotic cells can also exhibit significant immunostimulatory capacity [138, 139]. One other influential factor for these contradictory observations could be the timepoint of immunomonitoring: early assessment might miss immune activation and antitumor activity. Interestingly, clinical improvement could be recorded rather late after cryotreatment (up to 10 weeks) [140, 141] which is in line with the new concept that assessment of tumor response upon immunotherapy should be performed later than after conventional cytostatic therapy [142].

Table 3 only presents recent immunological observations of the past decade. Many observations reporting immunosuppression by cryoablation were made earlier and are discussed below, but are not presented in the table.

### 3.2. Cytokines and Stress Response

Unlike the other thermoablative methods, cryoablation induces a cytokine release syndrome (SIR—1–6.4% of all cases, with a mortality rate of 0.2–4%) [34, 35], assimilated to the *cryoshock phenomenon* which is clinically manifested by thrombocytopenia, disseminated intravascular coagulation (DIC), and pulmonary failure [35–38]. Cryoshock is mainly limited to ablation of hepatocytes [39]. In sheep and rats, the frequency of SIR correlated positively with the extent of cryoablated liver tissue, animals with more than 35% of ablated tissue presenting an elevated risk of SIR [37]. Moreover, cryoablation leads to significant increases of serum IFN*γ* TNF, IL-6, and IL-12, but not IL-10 within several hours after intervention [36, 83, 84]. In a rat model, cytokine release after cryoablation, RF ablation, and LITT was compared. Between 1 and 6 hours after cryoablation, significantly elevated serum levels of IL-6 were observed. IL-10 serum levels were slightly, but not significantly elevated [38]. In patients, TNF and IFN*γ* could remain elevated for up to four weeks [85]. In a model of transgenic mice overexpressing HSP-70 only a slight increase of HSP-70 expression could be observed which proved to be tissue-protective against cryonecrosis in skeletal muscle cells. Here, is has to be noted that no complete cryoablation, but only cryolesioning of skin and skeletal muscle was performed [88]. To our knowledge, no data on HSP expression after necrosis induction through cryoablation are available.

### 3.3. Antibodies

The earliest reports on immune modification after cryoablation described autoantibody production against ablated normal and tumor tissues in rabbits and monkeys, as well as in patients [41, 106–112, 114]. These antibodies were essentially IgG and IgM in the serum [41, 86, 114] and at the vicinity of the ablated lesion mainly IgG and IgA [115] appearing within two weeks after intervention [41, 86, 114]. In contrast, Müller and colleagues treated osteosarcoma in mice with cryoablation and found a decrease of tumor-binding antibodies [113]. 

Another effect of cryoablation was detected by Ravindranath and colleagues who observed a release of gangliosides into the circulation of CRC patients after cryoablation but not after RF ablation or surgery. At the same time, the group also described increasing titers of anti-ganglioside IgM antibodies [116]. Since anti-ganglioside antibodies have inhibitory effects on primary tumors, such as the induction of complement mediated killing [143] or apoptosis [144], production of antitumor antibodies might be one of the mechanisms underlying the immune-mediated tumor rejection following cryoablation [116].

### 3.4. Cellular Immunity

#### 3.4.1. Changes in Peripheral and Intratumoral Immune Cell Subsets

In rats, significantly elevated peripheral leukocyte counts—especially CD3^+^ and CD4^+^ T cells—were detectable between 1 and 14 days after intervention [38, 89]. In humans, cryoablation led to an increase of circulating T cells in few patients [99, 100]. In a randomized trial, cryoablation—compared to conventional surgery—led to increased numbers of helper T cells and activated T cells [101]. In a cohort of patients with liver metastases, an increase of the Th1/Th2 ratio was observed in the peripheral blood [84] whereas Zhou and colleagues reported a decrease of circulating CD4^+^CD25^+^Foxp3^+^T_reg_ after cryolesioning of HCC [105].

In tumor draining lymph nodes (TDLN), increased cellularity was observed both in T-cell (paracortical) and B-cell (germinal center) areas one week after treatment. Immunologic activity could remain increased over a time span of up to 10 weeks [90, 91]. Using a xenograft model of human melanoma in nude mice, Gazzaniga and coworkers further described a massive intravascular and peritumoral recruitment of leukocytes, essentially neutrophils and macrophages after cryoablation [86]. In a mouse mammary cancer model, the number of CD4^+^ T cells in TDLN was augmented. Interestingly, CD4^+^CD25^+^T_reg_ were more numerous after low rate freeze [79].

#### 3.4.2. Antitumor Specific Responses

Assessment of the immune modulation by cryoablation has yielded contradictory results. Older works have pointed out immunosuppressive effects: an increase of circulating immune effector cells was not of functional relevance for tumor rejection, rather, tumor outgrowth and increased metastasis was promoted. This indicated that cryoablation might mediate deleterious effects possibly by induction of suppressor T cells, today referred to as regulatory T cells, as well as delayed development of antitumor immunity [103, 104, 145]. In line with these findings, Machlenkin and colleagues did not observe cellular activation through cryotherapy as a monotherapy [92]. 

In contrast, other groups demonstrated immunologic activation in cryotreated animals (Table 3). Kimura and colleagues found an increased cytotoxic activity of peripheral lymphocytes and splenocytes against a murine leukemia virus-induced lymphoma [146]. Regression of distant metastases and resistance to tumor rechallenge was described by Bagley and colleagues who found that splenic lymphocytes isolated from sarcoma-bearing mice treated with cryoablation exhibited significantly increased cytotoxic activity against sarcoma cells as compared to those obtained from mice undergoing limb amputation [93]. Increased immunological activity could be delayed up to ten weeks after intervention [140].

Urano and colleagues observed an increased activity of tumor-specific cytotoxic T lymphocytes (CTL) seven days after cryoablation in a mouse colon-carcinoma model. These effects were only observed after ablation of a single nodule while ablation of several lesions abrogated immune-related tumor regression. Here, a threshold of ablated tissue volume that governed immune stimulation or suppression was proposed [94]. Interestingly, tumor-specific effector cells isolated from TDLN but not from the spleen or peripheral blood secreted a higher amount of IFN*γ* (between days 3 and 7 after treatment) as compared to cells obtained following surgical resection. 

In an OVA-expressing melanoma model, den Brok and colleagues observed an increase of antigen-loaded DC in draining lymph nodes both after cryoablation and RF-ablation. Of note, almost double as high cell numbers were observed compared to the induction through treatment with RF ablation [69]. Moreover, the numbers of infiltrating lymphocytes in TDLN were increased. These lymphocytes produced approximately 10-fold greater amounts of IFN*γ* upon stimulation with irradiated mammary adenocarcinoma cells after cryoablation than after surgical resection, delayed tumor growth and reduced the number of pulmonary metastases after adoptive transfer of TDLN cells of the cryoablated tumor [95]. The protection against a tumor rechallenge with B16-OVA cells was enhanced after cryoablation (50% surviving mice after 70 days) compared with the protective effect observed after RF ablation (20% surviving after 70 days) [79, 83, 95].

#### 3.4.3. Combination Therapies

While Machlenkin and colleagues observed no clinical benefit with cryoablation alone, the combination with an intratumoral injection of immature DC induced robust activation of CD4^+^ and CD8^+^ CTL [92]. This synergistic effect was further improved after pretreatment with anti-CD4 or anti-CD25 mAb for T_reg_ depletion [96]. 

In an OVA-expressing melanoma model, CTLA-4 blockade and depletion of regulatory T cells could further enhance cryoinduced tumor-specific T-cell responses [69]. Alternatively, concomitant injection of CpG 1668, a TLR 9 ligand, had a similar effect on T-cell recruitment. The route of adjuvant injection was crucial for immune induction, peritumoral CpG application showing to be superior to distant site. Although tumor growth was delayed after combination therapy, survival benefit was not superior to treatment with cryoablation alone [135].

Conditioning with cyclophosphamide injected one day before cryoablation led to increased IFN*γ*  production of tumor-antigen specific CD4^+^ T cells in a mouse colon cancer model as detected in intracellular cytokine staining, enhanced survival and even some complete remission. Three out of four animals cured with the combination therapy also survived a tumor rechallenge with no macroscopically visible tumor upon autopsy [97]. Moreover, adoptive transfer of spleen and lymph node cells from surviving mice led to an improved survival in tumor-bearing mice. Depletion experiments showed that CD8^+^ effectors were responsible for tumor elimination indicating that immunological memory had developed [97].

In the same OVA-expressing melanoma model used by den Brok and colleagues, Redondo and coworkers observed a clear survival advantage for mice treated with cryoablation combined with repeated topical application of imiquimod as an adjuvant indicating that TLR-7 activation can enhance tumor-specific immune responses induced by thermal treatment [98]. 

To sum up, all these reports suggest that combination of cryoablation with check point blockade or immunoadjuvants is a promising approach in the treatment of cancer patients.

### 3.5. Immune Response and Clinical Course

In patients with hormone refractory prostate carcinoma, a combination of cryoablation with injection of GM-CSF as adjuvant was evaluated. T-cell reactivity against autologous tumor tissue lysates as determined in IFN*γ*  ELISPOT was found to be weakly increased after therapy, and no correlation could be established between the breadth of the immune response and the clinical course as measured by analysis of PSA serum levels [85, 102]. Also in a small cohort of patients with RCC, increased cytotoxic T-cell activity and increased antitumor serum antibodies in selected patients were observed which only weakly correlated with a favorable clinical response. Here also, GM-CSF was applied as an adjuvant [87].

## 4. Microwave Ablation

As with RF ablation, microwave ablation therapy (MWA) induces hyperthermia leading to coagulative necrosis. The clinical application of MWA is, however, more limited than the thermoablative methods discussed above and only few groups have evaluated the immunomodulatory effects of MWA (Table 4).

### 4.1. Cytokines and Stress Response

MWA was described to induce HSP-70 expression in normal kidney tissue lysates obtained from treated rats, as detected with specific ELISA. However, HSP-70 expression was significantly lower upon MWA as compared to animals treated with RF ablation and cryoablation [117].

### 4.2. Cellular Immunity

In a mouse tumor model of HCC, only 2/10 animals experienced tumor rejection upon rechallenge after MWA, suggesting an existing but suboptimal protective antitumor immunity [118]. However, the protective effect could be improved by intratumoral coadministration of GM-CSF loaded microspheres, and even more by intraperitoneal CTLA-4 blockade. The triple combination not only led to rejection of newly inoculated tumors, but was also effective in the rejection of established distant tumors. Splenocytes isolated from the treated mice killed hepatoma cells *in vitro*, but not an unrelated tumor cell line. *In vitro* depletion experiments using mAb could further show that cytotoxicity was mediated by T cells (both CD4^+^ and CD8^+^) and NK cells, confirming that antitumor immunity was induced upon combination therapy [118].

One month after MWA, 10 patients with hypersplenism that had developed as a result of portal hypertension exhibited a transient peripheral increase of T helper cells (CD3^+^CD4^+^) and B cells, but not of cytotoxic (CD3^+^CD8^+^) T cells [22]. In a larger cohort of patients suffering from HCC, immune cell infiltration was studied by immunohistochemistry analyses of biopsy tumor samples taken either before or at different timepoints (3–30 days) after MWA application. A markedly increased infiltration of lymphocytes (predominantly CD3^+^ T cells, CD56^+^ NK cells, and macrophages, but not of B cells) was detected after MWA inside the ablated lesions, in the adjacent normal tissue and in distant untreated lesions [119].

### 4.3. Immune Response and Clinical Course

The density of infiltrates of lymphocytes, macrophages, and CD56^+^ cells into MWA-treated liver tissue correlated inversely with the risk of local recurrence [119]. 

Zhou and colleagues performed a phase I clinical study in ten HCC patients with chronic hepatitis B by combining local microwave tumor ablation with immunotherapy, which was applied at 3 timepoints, that is, on the day of the MWA and then on days 11 and 100. Immature and mature monocyte-derived DC loaded with autologous tumor lysate were injected into the rim between the ablation zone and normal liver parenchyma and into the groin lymph nodes, respectively. Additionally, *in vitro* activated lymphocytes were applied intravenously. A modest and transient effect on peripheral T-cell subsets (decrease of CD4^+^CD25^high^—possibly T_reg_—and increase of CD8^+^CD28^−^—differentiated CD8^+^ T cells—was reported one month after treatment concomitant with a reduction in hepatitis B virus load observed in some patients, but analyses of the antitumor specific responses were not performed in this study. Of note, this clinical setting does not allow determining whether the observed effects were due to the MWA itself, to the immunotherapy regimen or to the combination of both treatments [120].

## 5. High-Intensity Focused Ultrasound (HIFU)

In addition to mere hyperthermia, HIFU also exerts nonthermal mechanistic constraints (acoustic cavitation) on treated tissues that might contribute to and modulate its effects on the immune system [122] (Table 5). 

### 5.1. Cytokines and Stress Response

In breast cancer patients, increased HSP-70 expression was detected on the cell membrane of treated cancer cells. HSP expression was mainly found in the central necrosis zone while only a few positively stained cells were observed in the periphery [122].

### 5.2. Cellular Immunity

#### 5.2.1. Changes in Peripheral and Intratumoral Immune Cell Subsets

In patients with posterior uveal melanoma [125], pancreatic carcinoma [123], osteosarcoma, HCC, and RCC [126] that were treated with HIFU, increased percentages of CD4^+^ T cells and a higher CD4^+^/CD8^+^ ratio were observed [125, 126]. Another study observed only statistically significant higher NK cell percentages in the peripheral blood, while other leukocyte subsets remained stable [123].

In human breast cancer specimens collected 1-2 weeks after HIFU treatment, immunohistochemistry analyses showed a significant increase of T and B cells at the margin of the ablated region as compared to HIFU-untreated tumor samples. Interestingly, a subset of these cells were activated (CD57^+^) and expressed perforin and granzyme B, indicating the presence of activated cytotoxic effectors [27].

#### 5.2.2. Antitumor Specific Responses

In a model of experimental neuroblastoma, reduced secondary tumor growth after HIFU treatment was observed, although involvement of immune cells was not evaluated further [136]. 

Zhang and coworkers immunized mice with a vaccine consisting of a lysate of the H22 hepatoma cell line either untreated or pretreated *in vivo* with HIFU. Ten days after vaccination, animals received a subcutaneous tumor challenge. Tumor growth was significantly delayed in mice vaccinated with previously HIFU-treated tumor cells. However, survival was not different between the vaccination groups [121]. In another model, the same group utilized a DC vaccine loaded with cell debris from HIFU-treated or -untreated tumor cells. While tumor growth was again reduced, no survival advantage could be observed. However, increased activity of CD8^+^ splenocytes could be detected in IFN*γ* ELISPOT [124]. These were supported by *in vitro* experiments showing activation of bone-marrow derived DC upon incubation with tumor lysates and increased tumor killing by splenocytes harvested from HIFU-treated animals [121]. 

In contrast, several studies describe increased numbers of peripheral T cells following HIFU, which did not exert enhanced antitumor immunity [123, 125, 126].

Several groups observed a loss in tumor antigen expression in ablated prostate [23] or breast carcinoma [147, 148] lesions after HIFU. Such downregulation would be expected to lead to a reduced recognition of tumor tissue through antigen-specific T cells. Further investigations will be needed to determine whether the HIFU method is generally appropriate for efficient induction of antitumor T-cell immunity in patients.

## 6. Laser Ablation

Laser-induced thermotherapy (LITT) is applied widely for photocoagulation in retinal diseases, where the release of proinflammatory cytokines [127] or the activation of retina-specific T cells after panretinal photocoagulation (PRP) [149] have been described. In cancer patients, however, laser ablation is still experimental, and only scarce publications have addressed the modulation of cellular immunity through LITT (Table 6).

### 6.1. Cytokines and Stress Response

In patients, laser ablation led to increased levels of IL-6 and TNF-receptor 1 in the serum of patients suffering from primary and secondary malignant lesions of the liver 72 hours after treatment. Changes in the level of other proinflammatory cytokines such as TNF and IL-1*β* were not observed in this study [130]. 

In a murine model of colorectal liver metastases, LITT was also shown to enhance expression of HSP-70 at the margin of the coagulated tissue, with cytoplasmic and nuclear expression in sublethally damaged mouse hepatocytes and extraparenchymal cells. In tumor cells, this upregulation was detected between 12 hours and 7 days after intervention, with a peak at 24 hours [131].

### 6.2. Cellular Immunity

In WAG rats, Isbert and colleagues induced two independent tumors in the left and right liver lobes. One of the two tumors was either ablated with LITT or surgically removed, and immune cell infiltration into the untreated remaining tumor was compared to that observed in an untreated control group. Expression of CD8, CD86, MHC-class II, and adhesion molecules was found to be increased between 1 and 10 days after LITT at the tumor invasion front as compared to resection or no treatment, indicating an influx of immune cells [128]. Moreover, the growth of the untreated tumors was found to be considerably reduced in LITT-treated animals. 

Using a murine CRC model, Lin and coworkers observed an increased infiltration of CD3^+^ T cells into the tumor-host interface and into the tumor, as well as into the liver parenchyma and—to a certain extent—also into distant tumor lesions. Moreover, increased activation of splenocytes and tumor infiltrating lymphocytes was reported in *ex vivo* IFN*γ* ELISPOT without antigen restimulation [132]. Of note, these results were all obtained in animal models, and immune modulation after LITT has not been reported for cancer patients yet. 

Taken altogether, the available results strongly suggest that laser therapy, as shown for other thermoablation methods, can stimulate antitumor immune effector cells *in vivo *[128, 132].

## 7. Conclusion and Perspectives: Implications for Anticancer Therapy

During the past two decades, numerous publications in animal models and patients have shown that local thermoablative techniques can induce or enhance tumor-specific immune responses that contribute to tumor control. Although the sequential mechanisms involved are not yet fully elucidated, several pieces of the puzzle have been identified: thermal treatment induces necrosis and can (i) lead to local inflammation, release of danger signals—for example, heat shock proteins—which may even been detected systemically; (ii) stimulate the recruitment and activation of immune effector cells, including DC, at the vicinity and most probably inside the damaged tumoral tissue. Both processes occur rapidly, that is, within a few hours to days following intervention; (iii) activate antitumor adaptive immunity, including CD4^+^, CD8^+^ T cells, and antibody production which can contribute to local tumor elimination, control distant tumors including micrometastases, and establish long lasting antitumor immunological memory [69, 72, 83, 93, 97, 121]. The source of tumor-associated antigens for inducing specific T cells may be either necrotic dying cells [138, 150, 151] or sublethally damaged cells [62, 63]. Besides these direct mechanisms, the removal of tumor tissue leads to depletion of T_reg_ and more generally may overcome local immunosuppression shifting favorably the balance towards effective antitumor immunity [61, 96, 105].

Hence, thermoablation can trigger physiological cascades necessary and sufficient for a protective immune response. Obviously, several methods can be applied successfully, suggesting that the key element is the induction of local necrosis, which can be achieved by using different settings and temperatures. High temperatures (RF ablation, MWA, HIFU, and LITT) seem rather to sustain antitumor activity whereas both immunomodulatory and immunosuppressive effects have been reported upon cryoablation. Of note, the lesions induced by high-temperature thermoablation are probably not solely of necrotic nature but may also exhibit apoptotic cells [16, 152, 153]. Whether opposite immunological outcomes are hence related to a different balance between apoptosis, necrosis, and secondary necrosis, with apoptotic cells acting more in a tolerizing or immunosuppressive fashion and necrotic cells more immunogenic, is at the moment unclear [138, 139]. Because necrosis induction can be easily visualized during thermoablative intervention, controlled necrosis might be an ideal tool for inducing enhanced immunogenic cell death [154]. Interestingly, local hyperthermia between 40°C and 44°C has been also been described to modulate immunity, as reviewed elsewhere [155].

However, it should be noted that the reported effects of thermoablation alone on the immune system are generally modest, suggesting that such treatment as a monotherapy is in general not capable of inducing sufficient immune responses for full tumor protection [97, 118]. Thermotherapy should be therefore most effective in case of a limited tumor burden, ideally without detectable tumor postinterventionally, and not in advanced cancer where it is applied in most cases so far. Notably, combined therapies in order to enhance tumor-specific immune responses showed extremely promising results. Several strategies, such as check point blockade (anti-CTLA-4 mAb, T_reg_ depletion) [69] or application of adjuvants (interleukins or chemokines, GM-CSF, TLR agonists) have been evaluated in preclinical models but very little in clinical application yet. Randomized trials have still to be conducted. 

In summary, thermal ablation represents a promising component for cancer immunotherapy in the treatment of small or subclinical tumor lesions which can be attacked by the patient's immune system. By controlled induction of physiological stress, it offers the possibility of letting the “natural” immune response develop in its whole by breaking self-tolerance. So, thermal ablation of cancer provides a therapeutic implementation of the *danger model*. However, the induced antitumor immunity is weak and probably not sufficient alone to eradicate established tumors, but it can synergize with some chemotherapies and immunomodulating strategies. Selecting the appropriate thermoablative method and finding optimal combinations for individual patients will be an exciting challenge for the upcoming years. 

## Figures and Tables

**Table 1:** Thermal ablative methods in clinical use for the treatment of cancer and described effects on the immune system.

			Immune Modulation			
Treatment	Indication	Characteristics/principle	Component	Effect	Ref.	Species
Radiofrequency	*Clinical indication:* primary and	*Mechanism:* application of	Cytokines	+	[51–54]	Human
(RF) ablation	secondary malignancies in liver,	alternating RF current through tip	Danger	+	[55–61]	Animal
	kidney, lung, and bone	applicator placed around and in	signals	+	[62, 63]	Human
	[2, 8, 17, 49]	tumor tissue resulting in heat and	Granulocytes	+	[64, 65]	Animal
		coagulative necrosis			[54, 66]	Human
	*Experimental application:* tumors		NK cells	+	[61]	Animal
	of the breast [50]	*Approach:* percutaneous, open,		+	[67]	Human
		and intraoperative	Monocytes/	+	[66]	Animal
			Macrophages			
		*Image guidance:* US, CT, and MRI	DC	+	[68, 69]	Animal
					[51]	Human
			T cells*	+	[49, 57, 65, 68–74]	Animal
				+	[70, 75–78]	Human
			T_reg_	−	[54]	Human
			B cells	+	[75]	Human
			Antibodies*	+	[78]	Human

Cryoablation	*Clinical indication:* primary and	*Mechanism:* application of cold	Cytokines	+	[39, 83]	Animal
	secondary malignancies in liver,	through gaseous evaporation at		+	[36, 84, 85]	Human
	kidney, and prostate, as well as	the tip of a cryoprobe. Repetitive	Danger	?		
	dermatologic and ophthalmologic	freezing and thawing cycles lead to	signals			
	tumors [4, 33, 79, 80].	direct cellular damage through ice	Granulocytes	+	[86]	Animal
	*Experimental Application:* tumors	crystals, vascular and endothelial	NK cells	+	[83]	Animal
	of the breast [81].	injury, and eventually thrombosis		+	[87]	Human
		and ischemia [79, 82] resulting in	Monocytes/	+	[86, 88]	Animal
		coagulative necrosis and apoptosis	Macrophages			
		at the ablation margin	DC	+	[69]	Animal
						
						
		*Approach:* percutaneous, open,	T cells	+	[38, 69, 79, 83, 89–98]	Animal
		intraoperative		+	[85, 87, 99–102]	Human
			T_reg_	+	[103, 104]	Animal
				−	[105]	Human
		*Image guidance:* US, CT, and MRI	B cells	+	[91]	Animal
			Antibodies*	+	[86, 106–113]	Animal
				+	[41, 87, 114–116]	Human

Microwave	*Clinical indication:* mainly used for	*Mechanism:* application of	Cytokines	?	[117]	Animal
ablation therapy	treatment of HCC, but also other	microwaves through tip applicator	Danger	+		
(MWA)	primary and	leading to coagulative necrosis	signals			
	secondary	[21]	Granulocytes	?		
	malignancies of the liver [21, 22]		NK cells	+	[118]	Animal
		*Approach:* percutaneous, open,		+	[119]	Human
		and intraoperative	Monocytes/	+	[119]	Human
			Macrophages			
			DC	?		
		*Image guidance:* US, CT, and MRI	T cells	+	[118]	Animal
				+	[22, 119, 120]	Human
			T_reg_	?		
			B cells	+	[22]	Human
			Antibodies	?		

High-intensity	*Experimental application:* primary	*Mechanism:* application of focused	Cytokines	?		
focused	and secondary malignancies in	ultrasound beams of	Danger	+	[122]	Human
Ultrasound	breast, liver, pancreas, kidney,	high-intensity resulting in	signals			
(HIFU)	bone, prostate, and	coagulative necrosis	Granulocytes	?		
	soft-tissue-tumors [121]		NK cells	+	[123]	Human
			Monocytes/	?		
		*Approach:* noninvasive	Macrophages			
			DC	?		
		*Image guidance: *noninvasive	T cells	+	[121, 124]	Animal
		real-time US		+	[27, 125, 126]	Human
			T_reg_	?		
			B cells	+	[27]	Human
			Antibodies	?		

Laser induced	*Clinical indication:* broadly applied	*Mechanism: *placement of multiple	Cytokines	+	[130]	Human
thermotherapy	for photocoagulation in retinal	simultaneous fired laser fibers into	Danger signals	+	[131]	Animal
(LITT)	disease [127], primary, and	a tumor resulting in coagulative necrosis [129]	Granulocytes			
	secondary malignancies of the		NK cells	?		
	liver [128]					
	*Experimental application:* primary	*Approach:* percutaneous	Monocytes/	?		
	and secondary malignancies of the		Macrophages			
	breast, brain, bone, and prostate		DC	?		
	[129]	*Image guidance:* MRI, CT, and US	T cells	+	[128, 132]	Animal
			T_reg_	?		
			B cells	?		
			Antibodies	?		

Asterisks indicate allocation of T-cell or antibody responses to defined antigens.

Ref., reference number.

**Table 2:** Studies reporting immune modulation in cancer patients and animal models treated with RF ablation.

Species	Tumor	Model	Immunologic effect	References
Human	HCC (*n* = 1)		HSP-70, HSP-90 (cytoplasm, membrane)↑	[62]
	HCC (*n* = 8)		Activation of myeloid dendritic cells (blood)	[51]
			IL-1*β*, TNF (serum)↑	
	HCC (*n* = 20)		CD4^+^ and CD8^+^ cells (blood)↑	[70]
			CD3^−^CD56^+^, CD56^+^CD16^+^ cells (blood)↑	
			Activity of tumor-specific T cells↑	
	HCC (*n* = 37)		CD3^−^CD56^dim⁡^ cells (blood)↑	[67]
			Activity of CD3^−^CD56^dim⁡^ cells↑	
	HCC (*n* = 20)		Tumor-antigen specific T cells (blood)↑	[77]
	RCC (*n* = 6)		CD3^+^HLA-DR^+^, CD4^+^ and CD8^+^ cells (blood)↑	[71]
			CD56^+^CD16^+^cells (blood)↓	
	Liver metastases (*n* = 8)		Neutrophils (blood)↑	[66]
	HCC (*n* = 4)			
	Liver metastases (*n* = 6)		CD4^+^ and CD8^+^ cells (blood)↑	[76]
	HCC (*n* = 6)			
	Liver metastases of CRC		IL-6 (serum)↑	[52]
	(*n* = 10)			
	Liver metastases (*n* = 9)		IL-6 (serum)↑	[53]
	HCC (*n* = 2)			
	Liver metastases (*n* = 13)		CD4^+^ cells (blood)↓	[75]
	HCC (*n* = 4)		MUC-1 specific T cells (blood)↑	
			B cells (blood)↑ (only in metastatic cancer patients)	
			Trafficking of CD62L^+^ T cells into tissues	
	Lung metastases (*n* = 4)		IL-8, MIP-1*α*, MIP-1*β* (serum)↑	[54]
	NSCLC (*n* = 10)		IL-10 (serum)↑	
			CD4^+^CD25^+^Foxp3^+^ cells (blood)↓	
			Neutrophils (blood)↑	
	Liver metastases (*n* = 13)		IL-6 (serum)↑	[133]
	HCC (*n* = 4)		IL-10 (serum)↑	
	Metastases (*n* = 29)	± chemotherapy	CD4^+^ and CD8^+^ responses against tumor-specific	[78]
	Primary tumors (*n* = 26)		antigens (blood)↑	
	Metastases (*n* = 16)		Tumor-specific antibodies (serum)↑	
	HCC (*n* = 4)		HSP-70 (serum)↑	[63]
	RCC (*n* = 2)			

Mouse	CRC	CT26 hEpCam ± huKS-IL2	Antitumor activity (splenocytes)↑	[74]
(BALB/c)			Tumor growth (distant tumor)↓	
			Tumor growth (rechallenge)↓	
		C26	CD4^+^ cells (perinecrotic)↑	[65]
			Neutrophils (perinecrotic)↑	
			Neutrophils and lymphocytes (distant metastases)↑	
	HCC	BNL ± CCL3	CD11c^+^ cells (blood)↑	[73]
			CD11c^+^ cells (tumor)↑	
			CD4^+^ and CD8^+^ cells (tumor)↑	
			Tumor-specific cells (tumor)↑	
			Tumor growth (distant tumor)↓	
			Tumor growth (rechallenge)↓	

Mouse	Melanoma	B16-OVA ± CTLA4-mAb	CD8^+^ tumor-antigen specific T cells (blood)↑	[72]
(C57BL/6)		± T_reg_ depletion	Tumor growth (rechallenge)↓	
		B16-OVA ± CTLA4-mAb	Antigen loaded DC, DC maturation (draining	[69]
			lymphnodes)↑	
			Tumor growth (rechallenge)↓	
		B16-OVA ± DC	HSP-70, gp96 (tumor)↑	[57]
			HMGB1 (tumor)↑	
			CD8^+^ tumor-specific T cells (spleen, draining	
			lymph nodes)↑	
			Local recurrence↓	
			Tumor growth (rechallenge)↓	
	Urothelial carcinoma	MB49 ± DC	CD4^+^, CD8^+^ antitumor responses (splenocytes)↑	[49]
			CD11c^+^cells (tumor)↑	
			Tumor growth (rechallenge)↓	

Mouse	CRC	HT29	HSP-70 mRNA (cytoplasm)↑	[56]
(NIH (S)-nu)				

Rabbit	Hepatoma	VX2	Lymphocytes, plasma cells, and neutrophils	[68]
			(tumor)↑	
			Tumor-specific T cells (blood)↑	

Rat (Fisher)	Mammary	MatBIII	CD161^+^ cells (tumor-surrounding tissue)↑	[61]
			HSP-70 (tumor-surrounding tissue)↑	
		R3230 ± liposomal	HSP-70 (around central coagulation zone)↑	[59]
		doxorubicin		

Rat (rNU)	Hepatoma	SK-HEP-1	HSP-70, HSP-90 (cytoplasm, membrane)↑	[55]

**Table 3:** Recent Studies reporting immune modulation in cancer patients and animal models treated with cryoablation.

Species	Tumor	Model	Immunologic effect	References
Human	CRC (*n* = 110)		Gangliosides (GM_2_, GD_1a_, GT_1b_; serum)↑	[116]
			Antiganglioside antibodies (serum)↑	
	HCC (*n* = 111)		CD4^+^CD25^+^Foxp3^+^ cells (blood, ablation	[105]
			zone surrounding tissue)↓	
	Prostate (*n* = 20)		IFN*γ*↑, TNF (serum)↑	[85]
			Tumor-specific T-cell responses (blood)↑	
	Prostate (*n* = 12)	± GM-CSF	Tumor-specific T-cell responses (blood)↑	[85]
	RCC (*n* = 6)	+ GM-CSF	Tumor-specific T-cell responses (blood)↑	[87]
			Tumor-specific antibodies (serum)↑	
	Liver metastases		IL-6, TNF (serum)↑	[84]
	(*n* = 12)		Th1/Th2 ratio (blood)↑	
	CCC (*n* = 3)			

Mouse (BALB/c)	CRC	Colon-26 ± krestin	CD8^+^ antitumor T-cell reactivity (spleen)	[94]
			(↑)	
			Number of metastases↓	
		Colon-26 ± T_reg_	Tumor-specific CD8^+^ T cells (spleen)↑	[96]
		depletion ± DC + BCG	Tumor growth (distant tumors)↓	
		Colon-26 ±	Tumor-specific T cells (spleen, draining	[97]
		cyclophosphamide	lymph nodes)↑	
			Tumor growth (rechallenge)↓	
	Mammary	MT-901	IFN*γ*, IL-12 (serum)↑	[83]
			Tumor-specific T cells (draining	
			lymph nodes but not in spleen)↑	
			NK cell activity (spleen)↑	
			Tumor growth (rechallenge)↓	
			T cells (draining lymph nodes)↑	[95]
			Tumor-specific T cells (draining lymph	
			nodes)↑	
			Pulmonary metastases↓	
			Tumor-specific T cells (draining lymph	[79]
			nodes)↑	
			Pulmonary metastases (high-intensity	
			freezing)↓	
			Pulmonary metastases (low-intensity	
			freezing)↑	
	Melanoma	B16-OVA ± imiquimod	Tumor-specific T cell proliferation↑	[98]
			Tumor growth (rechallenge)↓	
		B16-MO5 ± DC	Tumor growth (rechallenge)↓	[92]

Mouse (C57BL/6)	Melanoma	B16-OVA ±	DC maturation and antigen uptake	[69]
		CTLA4-mAb	(TDLN)↑	
			Tumor growth (rechallenge)↓	
		B16-OVA ± CpG	DC (TDLN)↑	[135]
			CD4^+^, CD8^+^ T cells (TDLN)↑	
			OVA-specific T cells (TDLN)↑	
			Tumor growth (rechallenge after	
			peritumoral CpG administration)↓	

Mouse (NIH	Melanoma	IIB-MEL-J (human)	Neutrophils (RB6-5CG^+^)	[86]
(S)-nu)		± GM-CSF	(tumor-surrounding tissue)↑	
			macrophages (F4/80^+^; tumor-surrounding tissue)↑	
			DC (DEC205^+^; tumor-surrounding	
			tissue)↑	

Mouse (OT-I T cell	Lung	Lewis lung tumor	Tumor-specific CD8^+^ T-cell	[92]
receptor (V*α*2/V*β*5)		D122 ± DC	proliferation↑	
transgenic)			Th1 responses↑	
			Tumor growth (lung metastases)↓	

Rat (Wistar)	Glioma	C6	CD3^+^ and CD4^+^ T-cell percentages (blood)↑	[89]
			CD4^+^/CD8^+^ ratio (blood)↑	

(↑) Weak induction.

**Table 4:** Studies reporting immune modulation in cancer patients and animal models treated with MWA.

Species	Tumor	Model	Immunologic effect	References
Human	HCC (*n* = 82)		CD3^+^ cells, CD56^+^ cells (treated and distant tumors)↑	[119]
			CD68^+^ cells (treated and distant tumors)↑	
	HCC (*n* = 10)	± DC	Phase I study:	[120]
			CD4^+^CD25^high^ cells (blood)↓	
			CD8^+^CD28^−^ cells (blood)↑	

Mouse	HCC	Hepa 1–6	Activity of tumor-specific CD4^+^, CD8^+^ cells (spleen)↑	[118]
(C57BL/6)		±GM-CSF	NK1.1^+^ cells (spleen)↑	
		± CTLA4-mAb	Tumor growth (rechallenge)↓	

**Table 5:** Studies reporting immune modulation in cancer patients and animal models treated with HIFU.

Species	Tumor	Model	Immunologic effect	References
Human	Breast carcinoma (*n* = 23)		HSP-70 (membrane)↑	[122]
	Breast carcinoma (*n* = 48)		CD3^+^, CD4^+^, CD8^+^ cells (tumor)↑	[27]
			CD20^+^ cells (tumor)↑	
			CD57^+^ cells (tumor)↑	
	Pancreatic carcinoma (*n* = 15)		NK cells (blood)↑*	[123]
	Uveal melanoma (*n* = 5)		CD4^+^ cells (blood)↑	[125]
	Osteosarcoma (*n* = 6)		CD4^+^ cells (blood)↑	[126]
	HCC (*n* = 5)			
	RCC (*n* = 5)			

Mouse (Ajax)	Neuroblastoma	C1300 ± adriamycin	Tumor growth (rechallenge)↓	[136]

Mouse (C57BL/6J)	HCC	H22 ± DC	Activation of CD8^+^ cells (spleen)↑	[124]
			Tumor growth (rechallenge)↓	
		H22 ± tumor lysate vaccine	Cytolytic activity (spleen)↑	[121]
			Tumor growth (rechallenge)↓	

*NK cell phenotype was not specified.

**Table 6:** Studies reporting immune modulation in cancer patients and animal models treated with LITT.

Species	Tumor	Model	Immunologic Effect	References
Human	CRC (*n* = 4)		IL-6, TNF-R1 (serum)↑	[130]
	HCC (*n* = 3)			
	Other (*n* = 6)			

Mouse (CBA)	Liver metastases of CRC	MoCR	HSP-70 (cytoplasm, nuclear)↑	[131]
	Subcutaneous CRC tumors	MoCR	CD3^+^ cells (tumor-host interface)↑	[132]
			Spontaneous IFN*γ* production (spleen, lymph nodes, tumor,	
			and distant tumors)↑	

Rat (WAG)	CRC	CC531	CD8, CD86, MHC-II, CD11a, and ICAM1 expression (invasion	[128]
			front of distant tumors)↑	

## Abbreviations

CD: Cluster of differentiation
CRC: Colorectal carcinoma
CT: Computed tomography
CTLA: Cytotoxic T-lymphocyte antigen
DC: Dendritic cell
HCC: Hepatocellular carcinoma
HIFU: High-intensity focused ultrasound
HMGB1: High-mobility group protein B1
HSP: Heat shock protein
ICAM: Intercellular adhesion molecule
IFN: Interferon
IL: Interleukin
LITT: Laser-induced thermo therapy
mAb: Monoclonal antibody
MIP: Macrophage inflammatory protein
MRI: Magnetic resonance imaging
MWA: Microwave ablation therapy
n.a.: Not applicable
NSCLC: Nonsmall cell lung cancer
RCC: Renal Cell Carcinoma
RF: Radiofrequency
TDLN: Tumor draining lymph node
TLR: Toll-like receptor
TNF: Tumor necrosis factor
US: Ultrasound.
